# Associations between the built environment and physical activity in public housing residents

**DOI:** 10.1186/1479-5868-4-56

**Published:** 2007-11-12

**Authors:** Katie M Heinrich, Rebecca E Lee, Richard R Suminski, Gail R Regan, Jacqueline Y Reese-Smith, Hugh H Howard, C Keith Haddock, Walker S Carlos Poston, Jasjit S Ahluwalia

**Affiliations:** 1 Department of Public Health Sciences, University of Hawaii at Manoa, Honolulu, USA; 2 Department of Health and Human Performance, University of Houston, Houston, USA; 3 Kansas City University of Medicine and Biosciences, Kansas City, USA; 4 Department of Psychology, Castleton State College, Castleton, USA; 5 Department of Geography, American River College, Sacramento, USA; 6 Department of Basic Medical Science, School of Medicine, University of Missouri-Kansas City, Kansas City, USA; 7 Department of Medicine and Cancer Center, University of Minnesota, Minneapolis, USA

## Abstract

**Background:**

Environmental factors may influence the particularly low rates of physical activity in African American and low-income adults. This cross-sectional study investigated how measured environmental factors were related to self-reported walking and vigorous physical activity for residents of low-income public housing developments.

**Methods:**

Physical activity data from 452 adult residents residing in 12 low-income housing developments were combined with measured environmental data that examined the neighborhood (800 m radius buffer) around each housing development. Aggregated ecological and multilevel regression models were used for analysis.

**Results:**

Participants were predominately female (72.8%), African American (79.6%) and had a high school education or more (59.0%). Overall, physical activity rates were low, with only 21% of participants meeting moderate physical activity guidelines. Ecological models showed that fewer incivilities and greater street connectivity predicted 83% of the variance in days walked per week, *p *< 0.001, with both gender and connectivity predicting days walked per week in the multi-level analysis, *p *< 0.05. Greater connectivity and fewer physical activity resources predicted 90% of the variance in meeting moderate physical activity guidelines, *p *< 0.001, and gender and connectivity were the multi-level predictors, *p *< 0.05 and 0.01, respectively. Greater resource accessibility predicted 34% of the variance in days per week of vigorous physical activity in the ecological model, *p *< 0.05, but the multi-level analysis found no significant predictors.

**Conclusion:**

These results indicate that the physical activity of low-income residents of public housing is related to modifiable aspects of the built environment. Individuals with greater access to more physical activity resources with fewincivilities, as well as, greater street connectivity, are more likely to be physically active.

## Background

Despite widespread efforts to increase physical activity, less than half of Americans reach nationally recommended levels [1]. The relationship between neighborhood of residence and physical activity has become an important area of investigation based on initial findings suggesting that neighborhood of residence differentially influences physical activity rates [2]. When aggregated at the neighborhood level, socioeconomic status (SES) [3-6], access to physical activity resources [3,7], and the quality and accessibility of the pedestrian environment [7-9] appear to influence physical activity, although most investigations to date have typically relied on broadly-defined existing records (e.g., census data, business telephone listings) or self-reports of the presence or absence of neighborhood factors [10].

Several studies have found that residents in low SES neighborhoods report lower physical activity levels than residents of medium to high SES neighborhoods, even after adjusting for individual differences (e.g., income) [3-6]. This relationship has been hypothesized to reflect fewer physical activity resources and opportunities. In contrast, other studies have found increased walking (related to poverty and renting) [11] or overall energy expenditures [12] among those living in lower SES neighborhoods. Lower SES neighborhoods tend to have higher residential density, more renters (than owners), social norms of congregating outdoors, and more opportunities for energy expenditure through work or travel [11,12]. In addition, individuals in lower SES neighborhoods have lower rates of automobile ownership, increasing their reliance on public transit or non-motorized transportation modes [13].

Simply having more parks available has been found to facilitate walking and bicycling [14]. Lower-income urban adults with access to walking/jogging trails and parks have reported higher rates of physical activity (OR = 1.89 and 1.95, respectively) than those without trail and park access. As the number of available physical activity resources increased, so did the likelihood of meeting physical activity guidelines [3]. Nevertheless, low SES neighborhoods often have few physical activity resources available [6,7], and many are low quality or poorly maintained [15,16].

Neighborhood aesthetics also appear to influence physical activity. For example, the highest rates of resident walking are found in areas that are safe and aesthetically pleasing [8,9]. At the same time, physical decay, including "incivilities" (e.g., litter, vandalized buildings, graffiti), influences perceptions of neighborhood quality, impacting residents' health behaviors [11]. In neighborhoods with high rates of poverty and low rates of home ownership, the presence of incivilities may create settings that appear unappealing and unsafe, discouraging outdoor physical activity [11]. Brownson and associates found that lower-income individuals reported higher frequencies of incivilities (i.e., heavy traffic, unattended dogs, and air pollution) as barriers to physical activity than higher-income individuals, [17] demonstrating the complexity of understanding neighborhood influences on physical activity.

Walking, the most popular physical activity reported by Americans [11], may be especially sensitive to neighborhood conditions. Walking on a regular basis can result in significant health benefits, as moderate intensity physical activities equivalent to brisk walking (i.e., a daily, 30 minute brisk walk) help prevent numerous diseases and early death [5,6]. Despite the popularity and promising health benefits, few individuals get sufficient amounts of walking to gain health benefits [12]. In addition, the connectivity of streets, or the availability of direct and alternative travel routes between two destinations, fosters higher rates of resident physical activity for transportation, such as walking or bicycling [2,18]. Streets designed in a grid pattern with few obstacles (e.g., highways or other pedestrian barriers) preventing travel between destinations have high connectivity [2]. Street connectivity has been positively related to the number of minutes of moderate daily physical activity; neighborhood residents with high connectivity were 2.4 times more likely to meet walking recommendations than residents of neighborhoods with lower connectivity [19].

Previous research has used both self-reported perceptions and objective measurements of the environment to link elements of the built environment to physical activity [see [20-22] for reviews]. More researchers have studied self-reported perceptions of the environment than objective measures of the environment, and most studies on the built environment and physical activity have not directly assessed the environment by relying on pre-existing data sources [10]. Studies using objective and direct measures may be preferable over self-reported measures, because self-reported measures may reflect individual biases. Pre-existing data sources do not provide information about the modifiable characteristics of the environment, and direct objective measures can help identify these built environment elements that may be modified or enhanced via policy interventions [20,23]. There is a need to identify these modifiable characteristics of the environment that affect physical activity [24] using instruments that include concrete measures of the physical dimensions of the of the built environment factors under investigation [25]. Despite much interest and preliminary findings, many questions remain concerning the nature and scope of the relationship between the built environment and physical activity [26].

This study was designed to investigate how measured environmental factors (i.e., street connectivity and the number, type, accessibility, and quality of physical activity resources) are related to self-reported walking and vigorous physical activity for residents of low-income public housing developments. We hypothesized that residence in neighborhoods with higher street connectivity would be associated with more days walked per week and a greater likelihood of meeting guidelines for moderate physical activity. Further, we hypothesized that residence in neighborhoods with more physical activity resources and fewer incivilities would be associated with more days of vigorous physical activity.

## Methods

This project used cross-sectional data from two separate studies. Study 1: Pathways to Health (PATH), provided self-report physical activity data from residents residing in 12 public housing developments and section 8 housing (i.e., below poverty income levels); and Study 2: Understanding Neighborhood Determinants of Overweight and Obesity in Kansas City (UNDO-KC), provided street connectivity data and the number, type, accessibility, and quality of physical activity resources available in the neighborhoods surrounding the 12 public housing developments.

### Study 1

The PATH study held health fairs at 12 public housing developments in a large metropolitan area in order to recruit residents into a smoking cessation study [27-29]. All residents were eligible to attend.

#### Participants

Four hundred seventy people volunteered to participate in the 12 PATH health fairs (a participation rate of 18.6%; multiple methods were used to maximize participation rates, and are addressed in Heinrich et al. [16]). Mean age for participants was 44.0 y (*SD *= 16.6 y), and 338 of the participants (72.8%) were female. Over half of the participants (59.0%, *N *= 273) had the equivalent of a high school education or more. Most of the participants were African American (*N *= 367, 79.6%); 47 (10.2%) were Caucasian, 15 (3.3%) were Hispanic or Latino, 1 (0.2%) was Asian, and 31 (6.7%) were of other ethnic or racial groups not specified (for a complete report of all demographics by housing development, please see Table 1 in Heinrich et al. [16]). Housing development residents met the 2004 U.S. Department of Health and Human Service's poverty guidelines (i.e., annual household income of $18,850 or less per year for a family of four) [30].

**Table 1:** Ecological and physical activity characteristics of the neighborhoods (N = 12).

	**Characteristics**	**Mean**	**Standard deviation**	**Range**
**Ecological**	**Number of PA resources**	4.6	2.8	0 – 8
	**Street connectivity**	88.5	25.1	50 – 138
	**Accessibility (%)**	81.3	28.1	1.0 – 100.0
	**Average number of incivilities**	7.9	5.5	0.01 – 16.0
	**Average number of features used for PA**	2.5	1.1	0.01 – 4.2
	**Average feature quality**	2.3	0.9	0.01 – 3.0
	**Average number of visitor amenities**	3.5	1.4	0.01 – 5.0
	**Average amenity quality**	2.2	0.7	0.01 – 2.7
**Physical activity**	**Average days walked per week**	3.0	0.4	2.3–3.5
	**Average days of vigorous PA per week**	1.6	0.6	0.5 – 2.7
	**Percent meeting moderate PA guidelines**	20.4	8.1	11.1 – 36.0

PA = Physical activity

#### Measures

##### Self-reported physical activity

Two questions adapted from the National Health Interview Survey asked about walking: 1) "About how many days did you walk for exercise in the past two weeks?" and 2) "On average, how many minutes did you walk each time?" These questions have demonstrated a significant test-retest correlation in ethnic minority samples (*r *= 0.33, *p *< 0.05) [31]. Days of vigorous physical activity during the past week was determined by asking participants, "On how many of the last 7 days did you participate in any sports or exercise that made you sweat or breathe hard for at least 20 minutes at a time?" [32]. This question also has shown significant test-retest reliability with Kappa coefficients ranging from 60% to 84% [33]. Self-reported physical activity from the past seven days assessed using these questions has been found to have validity correlations of *r *= 0.50 and *r *= 0.53 with accelerometers [34].

#### Procedures

Health fairs were staffed by trained PATH team members (e.g., physicians, research assistants), trained Community Outreach Resident members, and local agencies (e.g., American Red Cross). Health fair participants visited ten stations in total and finished by eating a free healthy meal that emphasized high fruit and vegetable, and low fat consumption. Questionnaire data was verified by each interviewer and audited for completeness by a designated team member. To maintain database accuracy, data were double-entered.

### Study 2

The UNDO-KC study measured specific characteristics of the neighborhoods surrounding the PATH housing development locations, including the number and characteristics of physical activity resources and street connectivity. The UNDO-KC study's primary aim was to directly assess factors of the built environment thought to affect overweight and obesity [see [16]].

#### Neighborhoods

Neighborhoods were designated as an 800 m radius circle area around the center of each public housing development [35]. This distance was chosen to include all possible destinations within a 10 – 15 min walk for residents, and to include every physical activity resource to which a resident, even those in automobiles, might be exposed to on a daily basis.

#### Measures

The Physical Activity Resource Assessment instrument (PARA) was used to count and evaluate all neighborhood physical activity resources. The PARA, a checklist instrument, has shown good inter-rater reliability (*ks *> 0.77) and has been previously described in detail [15,16]. The PARA was used to measure the type of physical activity resource (e.g., park, trail, community center). The number of features used for physical activity was measured (13 possible features; e.g., tennis courts, baseball fields) along with the number of visitor amenities (12 possible amenities; e.g., benches, drinking fountains). The quality of each feature or amenity present was then objectively rated on a three point scale (i.e., 1 = poor, 2 = mediocre, 3 = good). The number of resource incivilities (12 possible incivilities; e.g., litter, graffiti) were also coded into one of four categories where 4 = not present, 3 = a little, 2 = a medium amount, and 1 = a lot, with operational definitions providing the cut points for these categories. Incivilities were reverse coded for analysis to determine the average number of incivilities per housing development. Cost for use was used to indicate physical activity resource accessibility (i.e., free = higher accessibility, pay = lower accessibility). A total of 55 physical activity resources were found for all neighborhoods (*M *= 4.6, *SD *= 2.78, per neighborhood), although one neighborhood had none. (See [16], Table 2 for complete PARA results by neighborhood.)

**Table 2:** Aggregated neighborhood-level correlations (*N *= 12).

	**Days walked per week**	**Percent meeting moderate PA guidelines**	**Days per week of vigorous PA**
**Number of PA resources**	-0.31	-0.30	0.43
**Connectivity**	0.74 **	0.87 **	-0.09
**Accessibility**	-0.11	0.14	0.58*
**Incivilities**	-0.62*	-0.38	0.47
**Features**	-0.01	0.12	0.36
**Feature quality**	0.02	0.04	0.49
**Amenities**	0.01	0.09	0.49
**Amenity quality**	-0.03	0.23	0.46

*p < 0.05;**p < 0.01

PA = Physical activity

To calculate street connectivity (i.e., intersection density), area maps were initially generated and verified during windshield surveys. Any misplaced streets were corrected or added as necessary to the maps. Then, two staff members examined all intersections and those containing three *or more *streets intersecting at the same point were counted to indicate street connectivity [18]. The counts ranged from 50 to 138 intersections of three *or more *streets with an average of 88.5 (*SD *= 25.1) per neighborhood.

### Statistical analyses

A total of 470 participants were entered into this combined study; however, walking and vigorous physical activity data were missing for 18 participants resulting in a sample size of 452. The average number of days walked per week, the status of meeting moderate physical activity guidelines, and the average days of vigorous physical activity per week were calculated for each individual.

Two conceptually different approaches were used to examine the association between housing development characteristics and physical activity outcomes. First, the association between housing development characteristics and population outcomes at the housing development level (*N *= 12) was examined. Next, outcomes at the participant level (*N *= 452) were explored using multi-level models. Specific methods used to develop both sets of models are outlined below.

#### Ecological models

In the first set of models, often termed Ecological Models, the association between housing project characteristics and the population average level of physical activity was examined [36,37].(Greenland, 2002; Wu Wen & Kramer, 1999). All individual and physical activity resource data were aggregated at the neighborhood level (*N *= 12), and the mean, standard deviation, and range values were determined for each variable. Ecological analysis is a powerful tool for answering questions about the population-wide effects of community interventions or characteristics.

Initially, Pearson product moment bivariate correlation analyses were computed between the aggregated individual variables (i.e., days walked per week, percent meeting moderate physical activity guidelines; i.e., ≥ 150 min/wk, and days per week of vigorous physical activity) and the environmental variables (i.e., number of physical activity resources, street connectivity, resource accessibility, total incivilities, number and quality of resource features, and the number and quality of resource amenities). To examine the population level effects, ecological analyses were performed through multiple regression modeling, again using neighborhood as the unit of analysis (N = 12). Three models were generated for each of the physical activity variables (dependents): 1) days walked per week; 2) percent meeting moderate physical activity guidelines; and 3) days per week of vigorous physical activity. Each model controlled for gender and used the eight ecological variables as the independent variables. All analyses were conducted using SPSS version 14.0 (SPSS Inc, Chicago, IL, USA), with alpha set *a prioi *at 0.05.

#### Multilevel models

Mixed regression models were used to examine the relationship between housing development characteristics and the three physical activity outcomes at the participant level. Two of the independent variables were not normally distributed. Days walked per week was categorized based on sample distribution into None (0, 29%), Low (0.5–2.5, 26%), Medium (3.0–6.0, 16%) and High (7.0, 29%), while days per week of vigorous physical activity was categorized based on sample distribution and meeting recommended levels into None (0, 62%), Low (1–2, 13%), Medium (3–5, 12%) and High (6–7, 13%). Because the physical activity outcome variables were non-Gaussian, each model was fit using the SAS GLIMMIX procedure.

In each model, housing development was included as a random factor along with the predictor variables. For the dichotomous outcome of meeting moderate physical activity guidelines, the model was fit using the binominal response distribution and the Logit link function. For the two ordinal outcomes, models were fit using the Multinominal (ordered) response distribution and the cumulative Logit link function. Thus, ordinal models examine the likelihood of a participant being at a higher level of the response variable given their score on an independent variable. Given the number of potential predictor variables and collinearity between variables, each predictor variable, along with gender, was screened prior to entry into a multivariate model [38]. In addition, because the individual level distributions of some key independent variables (e.g., number of physical activity resources, street connectivity, average number of incivilities per resource, average number of features per resource, average feature quality per resource, average number of amenities per resources, and average amenity quality per resource) were not normal, they were dichotomized using a median split. Accessibility also did not have a normal distribution and was dichotomized as either free or pay for use.

## Results

### Days walked per week

Of the 452 participants, 28.8% walked every day during the past week while 29.2% did not walk at all. Although men reported more walking (*M *= 3.65 days/wk, *SD *= 3.05) than women (*M *= 2.79 days/wk, *SD *= 2.79), this difference was not statistically significant, *p *> 0.05. The average number of days per week the participants reported walking was 3.03 (*SD *= 2.89; see Table 1). At the aggregated neighborhood level, the average number of days walked in the past week ranged from 2.3 to 3.5 days/wk (*M *= 3.0 days/wk, *SD *= 0.4 days/wk).

### Percent meeting moderate physical activity guidelines

Ninety-five participants (21.0%) indicated they walked an average of 30 minutes or more on five or more days per week (i.e., meeting recommended levels of ≥ 150 minutes per week). Some walking in the past week (0.5 to 4.5 days/wk) was reported by 36.7% (*N *= 166) of the participants, while almost one-third of the sample (29.2%, *N *= 132) reported no walking in the past two weeks. The average percent of participants meeting moderate physical activity guidelines at the neighborhood level ranged from 11.1% to 36.0% (*M *= 20.4%, *SD *= 8.1%; see Table 1).

### Days per week of vigorous physical activity

Sixty-two percent (*N *= 281) of participants reported that they did not do any vigorous physical activity during the past week (0 days/wk), while 12.8% (*N *= 58) were vigorously active every day (7 days/wk). Women reported slightly more vigorous physical activity (*M *= 1.58 days/wk, *SD *= 2.41) than men (*M *= 1.49 days/wk, *SD *= 2.55), but this difference was not statistically significant, *p *> 0.05. At the aggregated neighborhood level, days of vigorous physical activity performed during the past week ranged from 0.5 to 2.3 days (*M *= 1.6 days/wk, *SD *= 0.6 days/wk; see Table 1).

### Correlations between variables aggregated at the neighborhood level

Correlation analyses between the ecological and physical activity variables aggregated at the neighborhood level (*N *= 12) are presented in Table 2. Greater neighborhood street connectivity (*p *< 0.01) and fewer average incivilities per neighborhood (*p *< 0.05) were associated with more days walked per week. Higher street connectivity was also correlated with meeting moderate physical activity guidelines (*p *< 0.01). A greater percent of accessible physical activity resources was related to the number of days vigorous physical activity was performed during the past week (p < 0.05).

### Ecological regression analyses predicting physical activity variables

The number of neighborhood incivilities and street connectivity accounted for a significant amount (83.0%) of the variance in days walked per week (*p *= 0.001) (Table 3). Connectivity exerted slightly more influence than did incivilities on days walked per week (standardized *β *= 0.672 vs. -0.540 for connectivity and incivilities, respectively). Connectivity promoted while incivilities restricted the number of days walked per week.

**Table 3:** Multiple regression for days walked per week

*Days walked per week*			
**Model 1**	Standardized Beta	T value	p value

*Constant*	2.379	10.8	0.001
**Incivilities**	-0.540	-3.89	0.005
**Connectivity**	0.672	4.84	0.001

F = 21.8 (2,11); p < 0.001; R^2 ^= 0.83

PA = Physical activity

Connectivity also was associated with the percent of neighborhood residents meeting the moderate physical activity guidelines. Table 4 shows that street connectivity, along with the number of physical activity resources, accounted for 90.0% of the variance in the percent of neighborhood residents meeting guidelines for moderate physical activity. Neighborhoods with greater street connectivity and fewer physical activity resources had a higher percentage meeting moderate physical activity guidelines.

**Table 4:** Multiple regression for meeting moderate physical activity (PA) guidelines

*Percent meeting moderate PA guidelines*			
**Model 2**	Standardized Beta	t value	p value

*Constant*	-0.40	-0.12	0.909
**Connectivity**	0.902	8.40	0.001
**Number of PA resources**	-0.379	-3.53	0.006

F = 39.18 (2,11); p < 0.001; R^2 ^= 0.90

Physical activity resource accessibility was the only ecological variable related to days of vigorous physical activity per week (standardized *β *= 0.584). Greater accessibility (i.e., more free resources) was related to more days/wk of vigorous physical activity, and accounted for 34.0% of the variance in days/wk of vigorous physical activity (see Table 5).

**Table 5:** Multiple regression for days per week of vigorous physical activity (PA)

*Days per week of vigorous PA*			
**Model 3**	Standardized Beta	t value	p value

*Constant*	0.586	1.27	0.234
**Accessibility**	0.584	2.27	0.046

F = 5.17 (2,11); p < 0.05; R^2 ^= 0.34

### Multi-level model analysis predicting physical activity variables

An examination of the mixed model results showed that days walked per week were significantly predicted by the individual variable of male gender and the neighborhood variable of street connectivity. Females walked half as many days per week as males did (*Odds Ratio *= 0.43–0.91), while greater street connectivity resulted in 1–2 more days walked per week (*Odds Ratio *= 1.11–2.18). (See Table 6.) The percentage of participants meeting moderate physical activity guidelines was also significantly predicted by gender and street connectivity. This time, females were up to one-third less likely to meet moderate physical activity guidelines than were males (*Odds Ratio *= 0.37–0.98). Having greater street connectivity was linked to a 1.2 to 3.3 greater chance of meeting moderate physical activity guidelines (*Odds Ratio *= 1.21–3.26). (See Table 7.) There were no statistically significant predictors for days of vigorous physical activity per week (data not shown).

**Table 6:** Mixed model analysis for days walked per week

*Days walked per week*			
**Model 1**	Odds Ratio	95% CI	p-value

**Gender**	0.623	0.428 – 0.905	0.013
**Connectivity**	1.553	1.105 – 2.183	0.011

**Table 7:** Mixed model analysis for meeting moderate physical activity (PA) guidelines

*Percent meeting moderate PA guidelines*			
**Model 2**	Odds Ratio	95% CI	p-value

**Gender**	0.602	0.370 – 0.978	0.041
**Connectivity**	1.987	1.210 – 3.263	0.007

## Discussion

As hypothesized, higher street connectivity was associated with more days of walking per week and with meeting moderate physical activity recommendations in all three analyses. This is consistent with recent research and is important in efforts to reduce obesity, because residents who live in more connected areas are more likely to walk or bicycle for transportation [2,18,19]. In fact, highly connected neighborhoods have residents that are 2.4 times more likely to meet recommended levels of walking [19].

Contrary to the hypothesis, having more neighborhood physical activity resources or fewer incivilities was not related to more days of vigorous physical activity. It is possible that the residents were not using their neighborhood physical activity resources for their vigorous physical activity, but this usage information was not collected. Additionally, non-statistically significant variations in the number of physical activity resources existed between neighborhoods (*Range *= 0–8, *p *> 0.05), suggesting that the lack of findings might result, in part from having too few neighborhoods to detect an effect. Lee and her colleagues [39] have found that women who live in lower SES neighborhoods may get some physical activity benefit from having physical activity resources nearby, and Parks and associates [3] have previously shown a direct relationship between the number of available physical activity resources and the likelihood of meeting physical activity guidelines among urban, low-income residents. Correlation and multiple regression results did correspond with previous research indicating that residents who lived in neighborhoods with more freely accessible physical activity resources reported more vigorous physical activity [4]. However, days per week of vigorous physical activity were not significantly predicted by any individual- or neighborhood-level variable in the multi-level model.

The multi-level models did show the impact of gender on walking and meeting moderate physical activity guidelines, with males having significantly higher rates of each than females. Previous research with older adults has shown that males report more physical activity, while females report more involvement in household activities [40]. However, another study looking at adults of all ages found similar rates of males and females who report walking regularly at 33.7% and 33.6%, respectively [41]. It is possible that the 114 males who chose to attend the health fairs in this study had higher rates of walking than other males who lived in the housing developments, but this is impossible to determine.

Overall, the self-reported rates of moderate physical activity were low, with only 21% of the participants meeting recommendations. Similar low levels have been found for African American women [1]. Individuals from lower-income neighborhoods also have been found to have lower rates of physical activity [3,5,6]. Many of the participants walked at least some days (36.7%) or the recommended amount (21.0%) in the past week. This is not surprising, since walking is the most common type of physical activity [11]. The majority of the participants (62%) reported no vigorous physical activity and 74.8% did not meet recommended levels of vigorous physical activity. This was similar to the 2005 vigorous physical activity rates for Kansas and Missouri at 75% and 74.7%, respectively [42].

This study adds to the literature focusing on the relationship between the built environment and physical activity. Results help indicate which aspects of neighborhood quality are important in influencing behavior for low-income public housing residents. For example, the quality of features used for physical activity and visitor amenities was not significantly related with physical activity; however, more days of walking were reported in neighborhoods with fewer incivilities. Research by Giles-Corti and Donovan indicated that the quality of the environment may be more important than income level for increased walking [4], and incivilities are easier to improve than many other environmental variables through prompt maintenance by parks and recreation departments or private facilities.

One possible reason we did not find a significant relationship in the multi-level analyses between the number of physical activity resources per neighborhood and physical activity as Parks and associates [3] did may be the low number of physical activity resources in many neighborhoods, as numbers varied from zero to eight resources. This finding is similar to previous studies that have found few physical activity resources in low-income neighborhoods [6,7].

### Limitations of the study and directions for future research

Although we were able to comprehensively measure aspects of physical activity resources, there were additional environmental factors (e.g., steep terrain, poor lighting, and unsafe environments, crime) that were not included in this study. People living in areas with those characteristics have reported lower physical activity rates [6,43], and it is possible that these additional environmental factors might also influence physical activity among low-income housing residents. Future research could address this question.

The questions used to measure physical activity in this study did not ask about physically active transportation or lifestyle activities that may correlate differently with the environment than leisure time physical activity. In addition, participants were not asked where they completed their physical activity. Future research should examine all of these variables to determine the exact extent of the impact of street connectivity by purpose of the physical activity. While street connectivity by road design is not easy to change, creating "cut-through" paths between cul-de-sacs or dead-end streets can increase connectivity for pedestrians, possibly resulting in increased non-motorized transportation or increased opportunities for walking.

The design of this study was cross-sectional and results cannot be used to indicate causal relationships. Future research could employ longitudinal designs that track environmental changes and changes in physical activity levels. This would help elucidate the specific environmental characteristics with the best capability of causing behavior change.

Since all participants in this study were low-income residents of public housing developments, it is possible that more information could be gleaned by including participants from other SES levels. Future comparisons should be made with medium and high SES neighborhoods as they have been found to have more resources and residents with higher rates of physical activity [7]. It is possible that comparisons could illuminate built environment differences that vary between SES levels.

## Conclusion

This study identified modifiable aspects of the built environment that affected physical activity rates for low-income residents of public housing developments. Results of this study should be interpreted in light of the respective analyses, showing the importance of street connectivity, average incivilities, the number of physical activity resources, and resource accessibility (i.e., cost) as significant predictors of physical activity at the neighborhood level. At the more conservative, individual level, only street connectivity and male gender remained as statistically significant predictors for walking and meeting moderate physical activity guidelines. The use of comprehensive study approaches such as this are consistent with social ecological models (highly recommended for their global effects) and should provide further insight into how physical activity is influenced by environmental characteristics [44]. Those responsible for public policy can facilitate health promotion by focusing on these modifiable characteristics of the built environment at the appropriate level to create opportunities and remove barriers for physical activity. Future research could examine additional aspects of the built environment that were not included in this study, as well as make comparisons to areas of other SES levels.

## Abbreviations

PATH = Pathways to Health

UNDO-KC = Understanding Neighborhood Determinants of Obesity – Kansas City Study

PARA = Physical Activity Resource Assessment;

## Competing interests

The author(s) declare that they have no competing interests.

## Authors' contributions

KMH primarily wrote the manuscript and helped with the collection of both data sets. REL provided the environmental data and intensive guidance through all phases of the manuscript development and writing. RS provided guidance with conducting the statistical analysis and writing the manuscript. GRR and JYR provided guidance for writing the manuscript and helped with the collection of both data sets. HHH provided geographic support and assisted with environmental variable construction. CKH provided guidance with the statistical analysis. WSCP provided guidance for writing the manuscript. JSA provided the individual-level data and guidance for writing the manuscript. All authors read and approved the final manuscript.

## References

[B1] Macera CA, Ham SA, Yore MM, Jones DA, Ainsworth BE, Kimsey CD, Kohl HW (2005). Prevalence of physical activity in the United States: Behavioral Risk Factor Surveillance System, 2001. Prev Chronic Dis.

[B2] Saelens BE, Sallis JF, Frank LD (2003). Environmental correlates of walking and cycling: Findings from the transportation, urban design, and planning literatures. Ann Behav Med.

[B3] Parks SE, Housemann RA, Brownson RC (2003). Differential correlates of physical activity in urban and rural adults of various socioeconomic backgrounds in the United States. J Epidemiol Community Health.

[B4] Giles-Corti B, Donovan RJ (2002). Socioeconomic status differences in recreational physical activity levels and real and perceived access to a supportive physical environment. Prev Med.

[B5] Kavanagh AM, Goller JL, King T, Jolley D, Crawford D, Turrell G (2005). Urban area disadvantage and physical activity: a multilevel study in Melbourne, Australia. J Epidemiol Community Health.

[B6] Wilson DK, Kirtland KA, Ainsworth BE, Addy CL (2004). Socioeconomic status and perceptions of access and safety for physical activity. Ann Behav Med.

[B7] Estabrooks PA, Lee RE, Gyurcsik NC (2003). Resources for physical activity participation: Does availability and accessibility differ by neighborhood socioeconomic status?. Ann Behav Med.

[B8] Cervero R, Duncan M (2003). Walking, bicycling, and urban landscapes: Evidence from the San Francisco bay area. Am J Public Health.

[B9] Troped PJ, Saunders RP, Pate RR, Reininger B, Ureda JR, Thompson SJ (2001). Associations between self-reported and objective physical environmental factors and use of a community rail-trail. Prev Med.

[B10] McCormack G, Giles-Corti B, Lange A, Smith T, Martin K, Pikora TJ (2004). An update of recent evidence of the relationship between objective and self-report measures of the physical environment and physical activity behaviours. J Sci Med Sport.

[B11] Ross CE, Mirowsky J (2001). Neighborhood disadvantage, disorder, and health. J Health Soc Behav.

[B12] Lee RE, Cubbin C, Winkleby M (2007). Contributions of neighborhood SES and physical activity resources to physical activity in women. J Epidemiol Comm Health.

[B13] Bhat CR, Guo JY (2007). A comprehensive analysis of built environment characteristics on household residential choice and auto ownership levels. Transport Res Part B.

[B14] Zlot AI, Schmid TL (2005). Relationships among community characteristics and walking and bicycling for transportation or recreation. Am J Health Promot.

[B15] Lee RE, Booth K, Reese-Smith J, Regan G, Howard H (2005). The physical activity resource assessment instrument: evaluating features, amenities, and incivilities of physical activity resources in urban neighborhoods. Int J Behav Nutr Phys Act.

[B16] Heinrich KM, Lee RE, Regan GR, Reese-Smith JY, Howard HH, Haddock CK, Poston WSC, Ahluwalia JS How does the built environment relate to BMI and obesity prevalence among public housing residents?. Am J Health Promot.

[B17] Brownson RC, Baker EA, Housemann RA, Brennan LK, Bacak SJ (2001). Environmental and policy determinants of physical activity in the United States. Am J Public Health.

[B18] Handy SL, Boarnet MG, Ewing R, Killingsworth RE (2002). How the built environment affects physical activity: Views from urban planning. Am J Prev Med.

[B19] Frank LD, Schmid TL, Sallis JF, Chapman J, Saelens BE (2005). Linking objectively measured physical activity with objectively measured urban form: Findings from SMARTRAQ. Am J Prev Med.

[B20] Sallis JF, Bauman A, Pratt M (1998). Environmental and policy interventions to promote physical activity. Am J Prev Med.

[B21] Sallis JF, Owen N, Glantz K, Rimer BK, Lewis FM (2002). Ecological models of health behavior. Health behavior and health education.

[B22] Humpel N, Owen N, Leslie E (2002). Environmental factors associated with adults' participation in physical activity: a review. Am J Prev Med.

[B23] Booth KM, Pinkston M, Poston WSC (2005). Obesity and the built environment. J Am Diet Assoc.

[B24] Suminski RR, Poston WS, Petosa RL, Stevens E, Katzenmoyer LM (2005). Features of the neighborhood environment and walking by U.S. adults. Am J Prev Med.

[B25] Northridge ME, Sclar ED, Biswas P (2003). Sorting out the connections between the built environment and health: a conceptual framework for navigating pathways and planning healthy cities. J Urban Health.

[B26] Popkin BM, Duffey K, Gordon-Larsen P (2005). Environmental influences on food choice, physical activity and energy balance. Physiol Behav.

[B27] Okuyemi KS, James AS, Mayo MS, Nollen N, Catley D, Choi WS, Ahluwalia JS (2007). athways to Health: A cluster randomized trial of nicotine gum and motivational interviewing for smoking cessation in low-income housing. Health Educ Behav.

[B28] Pulvers KM, Catley D, Okuyemi K, Scheibmeir M, McCarter K, Jeffries SK, Ahluwalia JS (2004). Gender, smoking expectancies, and readiness to quit among urban African American smokers. Addict Behav.

[B29] Ahluwalia JS, Nollen N, Kaur H, James AJ, Mayo M, Resnicow K (2007). Pathways to Health: Cluster-randomized trial to increase fruit and vegetable consumption among smokers in public housing. Health Psychol.

[B30] United States Department of Health and Human Services, Office of the Secretary (2004). The 2004 HHS poverty guidelines.

[B31] Rauh MJD, Hovell MF, Hofstetter CR, Sallis JF, Gleghorn A (1992). Reliability and validity of self-reported physical activity in Latinos. Int J Epidemiol.

[B32] Douglas K, Collins JL, Warren C, Kann L, Gold R, Clayton S, Ross JG, Kolbe LJ (1997). Results from the 1995 National College Health Risk Behavior Survey. J Am Coll Health.

[B33] Brener ND, Collins JL, Kann L, Warren CW, Williams BI (1995). Reliability of the youth risk behavior survey questionnaire. Am J Epidemiol.

[B34] Sallis JF, Haskell WL, Wood PD, Fortmann SP, Rogers T, Blair SN, Paffenbarger RS (1985). Physical activity assessment methodology in the five city project. Am J Epidemiol.

[B35] Porter DE, Kirtland KA, Neet MJ, Williams JE, Ainsworth BE (2004). Considerations for using a geographic information system to assess environmental supports for physical activity. Prev Chronic Dis.

[B36] Greenland S (2002). A review of multilevel theory for ecologic analyses. Stat Med.

[B37] Wu Wen S, Kramer MS (1999). Uses of ecologic studies in the assessment of intended treatment effects. J Clin Epidemiol.

[B38] Hosmer DW, Lemeshow S (2000). Applied logistic regression.

[B39] Lee RE, Cubbin C, Winkleby M (2007). Contribution of neighbourhood socioeconomic status and physical activity resources to physical activity among women. J Epidemiol Community Health.

[B40] Lee YS (2005). Gender differences in physical activity and walking among older adults. J Women Aging.

[B41] Eyler AA, Brownson RC, Bacak SJ, Housemann RA (2003). The epidemiology of walking for physical activity in the United States. Med Sci Sports Exerc.

[B42] Centers for Disease Control and Prevention Behavioral Risk Factor Surveillance System: 2005 Kansas vs Missouri Physical Activity.

[B43] Saelens BE, Sallis JF, Black JB, Chen D (2003). Neighborhood-based differences in physical activity: an environmental scale evaluation. Am J Public Health.

[B44] Spence JC, Lee RE (2003). Toward a comprehensive model of physical activity. Psychol Sport Exerc.

